# Multigene Sequence-Based and Phenotypic Characterization Reveals the Occurrence of a Novel Entomopathogenic Nematode Species, *Steinernema anantnagense* n. sp.

**DOI:** 10.2478/jofnem-2023-0029

**Published:** 2023-07-13

**Authors:** Aashaq Hussain Bhat, Ricardo A. R. Machado, Joaquín Abolafia, Tarique Hassan Askary, Vladimír Půža, Alba Nazaret Ruiz-Cuenca, Aasha Rana, Samy Sayed, Laila A. Al-Shuraym

**Affiliations:** Department of Biosciences and University Center for Research and Development, Chandigarh University, Gharuan, Mohali, Punjab, 140413, India; Experimental Biology Research Group, Institute of Biology, Faculty of Sciences, University of Neuchâtel, Neuchâtel, Switzerland; Departamento de Biología Animal, Biología Vegetal y Ecología, Universidad de Jaén, Campus “Las Lagunillas”, Jaén, Spain; Division of Entomology, Faculty of Agriculture, Sher-e-Kashmir University of Agricultural Sciences and Technology of Kashmir, Wadura Campus, Jammu and Kashmir, India; Biology Centre of the Czech Academy of Sciences, Institute of Entomology, Branišovská 31, 37005 České Budějovice, Czech Republic; Department of Zoology, Faculty of Basic and Applied Sciences, Madhav University, Pindwara (Sirohi), Rajasthan, 307026, India; Department of Economic Entomology and Pesticides, Faculty of Agriculture, Cairo University, 12613, Giza, Egypt; Department of Biology, College of Science, Princess Nourah Bint Abdulrahman University, P.O. Box 84428, Riyadh 11671, Saudi Arabia

**Keywords:** Species description, *Steinernema* nematodes, molecular characterization, phylogenetic systematics, *Xenorhabdus*, taxonomy, biological control, sustainable agriculture

## Abstract

Three entomopathogenic nematode populations were isolated from agricultural fields in the Anantnag district of Jammu and Kashmir (India). Sequences of multiple gene regions and phenotypic features show that they are conspecific and represent a novel species. Molecular and morphological features provided evidence for placing the new species into the “*Kushidai*” clade. Within this clade, analysis of sequence data of the internal transcribed spacer (ITS) gene, the D2D3 region of the 28S rRNA gene, the mitochondrial cytochrome oxidase I (*mtCOI*) gene, and the mitochondrial 12S (*mt12S*) gene depicted the novel species as a distinctive entity closely related to *Steinernema akhursti*, *S. kushidai*, and *S. populi*. Phylogenetic analyses also show that the new species is a sister species to *S. akhursti*, and these two species are closely related to *S. kushidai* and *S. populi*. Additionally, the new species does not mate or produce fertile progeny with any of the closely related species, reinforcing its uniqueness from a biological species concept standpoint. The new species is further characterized by the third-stage infective juveniles with almost straight bodies (0.7–0.8 mm length), poorly developed stoma and pharynx, and conoid-elongate tail (49–66 µm) with hyaline posterior part. Adult females are characterized by short and conoid tails bearing a short mucron in the first generation and long conoid tails with thin mucron in the second generation. Adult males have ventrally curved spicules in both generations. Moreover, the first-generation male has rounded manubrium, fusiform gubernaculum, conoid and slightly ventrally curved tails with minute mucron, and the second generation has rhomboid manubrium anteriorly ventrad bent, and tails with long and robust mucron. The morphological, morphometrical, molecular, and phylogenetic analyses support the new species status of this nematode, which is hereby described as *Steinernema anantnagense* n. sp. The bacterial symbiont associated with *S. anantnagense* n. sp. represents a novel species, closely related to *Xenorhabdus japonica*. These findings shed light on the diversity of entomopathogenic nematodes and their symbiotic bacteria, providing valuable information for future studies in this field.

Entomopathogenic nematodes (EPNs) of the genus *Steinernema* (Travassos, 1927) are among the most important biological control agents used in agriculture to control insect pests. The nematodes of this genus are associated with entomopathogenic bacteria of the genus *Xenorhabdus*, carried in a specialized receptacle structure hosted in the digestive tract of the free-living infective juveniles (IJs) (Chaston et al., 2011). The infective juveniles search for insects, and once inside the hosts, they release their bacterial symbiont into the hemocoel. Bacteria kill the insect hosts via toxins, enzymes, and insecticidal compounds produced during bacteria multiplication, making these symbiotic organisms highly valuable pest management tools in sustainable and eco-friendly agriculture.

The genus *Steinernema* is wider in terms of the number of species, when compared to other known EPN genera, with more than 125 valid species that have been described from different geographical regions, except Antarctica (Hominick et al., 1996; Bhat et al., 2020a; Machado et al., 2022; Malan et al., 2023). On the basis of the sequences of the internal transcribed spacer (ITS) region of the rRNA, the species of the genus *Steinernema* have been phylogenetically divided into 12 multiple species clades: “*Affine*”, “*Bicornutum*”, “*Cameroonense*”, “*Carpocapsae*”, “*Costaricense*”, “*Feltiae*”, “*Glaseri*”, “*Karii*”, “*Khoisanae*”, “*Kushidai*”, “*Longicaudum*” and “*Monticola*”; and three monospecies clades: *S. neocurtillae, S. unicornum*, and *S. rarum* (Spiridonov et al., 2016). The “*Kushidai*” clade currently contains three species, which are: *S. kushidai* (Mamiya, 1988), *S. akhursti* (Qiu et al., 2005), and *S. populi* (Tian et al., 2022), which are characterized by the average size of the IJs (body length of 700–1000 µm).

The diversity of the genus *Steinernema* reported in India is apparently very high (Bhat et al., 2020a), with 14 species isolated from Indian soils (Bhat et al., 2021a) from more than 125 valid *Steinernema* species. The *Steinernema* species that have been isolated from the Indian subcontinent include four from the “*Bicornutum*” clade: *S. bicornutum* (Hussaini et al., 2001), *S. riobrave* (Ganguly et al., 2002), *S. pakistanense* (Bhat et al., 2018), and *S. abbasi* (Bhat et al., 2021a); four from the “*Carpocapsae*” clade: *S. carpocapsae* (Hussaini et al., 2001), *S. tami* (Hussaini et al., 2001), *S. surkhetense* (Bhat et al., 2017), and *S. siamkayai* (Bhat et al., 2021b); three from the “*Glaseri*” clade: *S. sangi* (Yadav et al., 2012), *S. indicum* (Patil et al., 2023) and *S. hermaphroditum* (Bhat et al., 2019); and three from the “*Glaseri*” clade: *S. sangi* (Lalramnghaki et al., 2017), *S. cholashanense* (Mhatre et al., 2017), and *S. feltiae* (Askary et al., 2020). In addition, three species, *S. thermophilum*, *S. meghalayense*, and *S. dharanai*, were synonymized with already existing species: *S. abbasi, S. carpocapsae*, and *S. hermaphroditum*, respectively (Ganguly & Singh, 2000; Ganguly et al., 2011; Kulkarni et al., 2012; Hunt & Subbotin, 2016). The following species were declared species *inquirendae*: *S. masoodi, S. seemae, S. qazi*, and *S. sayeedae* (Ali et al., 2005; Ali et al., 2009; Ali et al., 2010; Ali et al., 2011; Hunt & Subbotin, 2016). Ganguly et al. (2002) also reported finding *S. riobrave* Cabanillas, Poinar & Raulston, 1994 but the identification was made based only on few morphometrical characters and the finding of *S. riobrave* in India is thus doubtful. Previously, no novel species of the family Steinernematidae have been reported from India so far (Bhat et al., 2020a; Rana et al., 2020; Bhat et al., 2021b; Askary et al., 2022), but recently one new species namely *Steinernema indicum* (Patil et al., 2023) has been added in it.

In order to characterize the prevalence and distribution of EPNs in Indian soils, a survey was conducted in the Pir Panjal Range, in the Kashmir region of the Indian subcontinent. As a result of this survey, several nematode populations were recovered, including three isolates: Steiner_6, Steiner_7, and Steiner_8. Initial molecular characterization suggests that these three isolates are conspecific and represent a new species in the genus *Steinernema*. In this study, we describe *Steinernema anantnagense* n. sp. based on morphological observations and morphometric analysis using light microscopy (LM) and scanning electron microscopy (SEM), as well as molecular studies based on genetic sequences of ribosomal RNA and mitochondrial genes. Self-crossing and cross-hybridization experiments were also used. In addition, we isolated and characterized the symbiotic bacterium associated with *S. anantnagense* n. sp.

## Materials and Methods

### Nematode survey and collection

*Steinernema anantnagense* n. sp. Steiner_6, Steiner_7, and Steiner_8 nematodes were isolated from soil samples collected in the Pir Panjal Range of Kashmir Valley, India using *Corcyra cephalonica* Stainton (Lepidoptera: Pyralidae) larvae as a bait insect. The isolates Steiner_6, Steiner_7, and Steiner_8 were collected in the Waghama area of Bijbehara Anantnag of the union territory of Jammu and Kashmir (GPS coordinates: 33.828914, 75.100091; 1606 m above the sea level) from soils around roots of willow, walnut, and apple intercrops, respectively, in areas adjoining district Anantnag, India. The insect cadavers recovered from soil samples were washed with ddH_2_O, sterilized with 0.1% NaOCl_2_, and nematode IJs were recovered from them by the White trap method (White, 1927). The IJs were sterilized with 0.1% NaOCl_2_ and stored in 250 mL tissue culture flasks in Biological Oxygen Demand incubator at 15°C. The new species has been registered in the ZooBank at urn:lsid:zoobank.org:pub:210D5242-2C15-437F-8D57-B00EECD98B85.

### Morphological and morphometrical characterization

Different life stages of *S. anantnagense* n. sp. were obtained from infected *Galleria mellonella* larvae exposed to 100 IJs/insects in a 15 cm-diameter Petri dish lined with moistened Whatman number 1 filter paper and kept in the dark at 25°C. The wax moth larvae died within 48 h after inoculation. After they died, the insect cadavers were transferred to a modified White trap (Kaya & Stock, 1997) and incubated at 25°C until IJs emerged. First- and second-generation adult nematodes were obtained by dissecting infected *G. mellonella* cadavers in Ringer’s solution after 3–4 and 6–7 days of infection, respectively. Infective juveniles (IJs) were collected after they emerged from *G. mellonella* cadavers in White traps (White, 1927). Nematodes were killed with water at 60°C, fixed in 4% formalin solution (4 mL formaldehyde, 1 mL Glycerol, 95 mL ddH_2_O), dehydrated by the Seinhorst method (Seinhorst, 1959), and transferred to anhydrous glycerin. Nematodes were, after that, picked with a peacock feather and mounted on permanent glass slides with extra layers of paraffin wax to prevent the flattening of the nematodes as described (Bhat et al., 2022). Morphometric measurements were taken using the Nikon DS-L1 image acquisition software mounted on a phase-contrast microscope (Nikon Eclipse 50i) in μm. Light microscopy photographs were captured using a Nikon Eclipse 80i microscope (Olympus, Tokyo, Japan) equipped with differential interference contrast optics (DIC) and a Nikon Digital Sight DS-U1 camera. For the scanning electron microscopy (SEM), nematodes preserved in 4% formalin were re-hydrated in distilled water, dehydrated in a graded ethanol-acetone series, critical point dried with liquid CO_2_, mounted on SEM stubs with a carbon tape, coated with gold in sputter coater, and observed with a Zeiss Merlin microscope (5 kV) (Zeiss, Oberkochen, Germany) (Abolafia, 2015). All micrographs were processed using Adobe® Photoshop® CS. Morphological characters of closely related species were taken from the original publications. The terminology used for the morphology of stoma and spicules follows the proposals by De Ley et al. (1995) and Abolafia and Peña-Santiago (2017a), respectively, and the terminology for pharynx follows the proposals by Bird and Bird (1991) and Baldwin and Perry (2004).

### Self-crossing and cross-hybridization experiments

Self-crossing and cross-hybridization experiments were carried out using *G. mellonella* larvae hemolymph as described by Kaya & Stock, 1997 with minor modifications. To this end, drops of hemolymph obtained from surface-sterilized *G. mellonella* larvae were placed in sterile Petri dishes (35×10 mm). Hemolymph drops were treated with a small amount of phenylthiourea to prevent melanization. Then 40–60 surface-sterilized IJs (0.1% NaOCl for 30 min, followed by thrice rinse through sterile distilled water) were added to the hemolymph drops. Then, Petri dishes were wrapped in moistened tissue paper and kept in plastic bags at 25°C (room temperature). Petri dishes were observed daily for the presence of males and virgin females. Then, males and virgin females in the ratio of 3:3 were placed separately in fresh hemolymph drops and were crossed with adults of the opposite sex of the other species. Controls consist of crosses of identical isolates; some females were kept without males to check their virginity (n=30). The Petri dishes were observed daily for 15 days to determine the production of offspring. Experiments were conducted twice under the same conditions. The following species were crossed: *Steinernema anantnagense* n. sp. (Steiner_6, Steiner_7, and Steiner_8), *S. ichnusae Sardinia*, *S. litorale* Aichi, *S. weiseri*, *S. akhursti* Akh, *S. citrae*, *S. cholashanense* GARZE, *S. feltiae* P1, *S. silvaticum*, *S. africanum* RW14-M-C2a-3, and *S. xueshanense* DEQ.

### Nematode molecular characterization and phylogenetic analyses

Genomic DNA was extracted from single virgin females as described (Bhat et al., 2023). Briefly, several virgin females were first washed with Ringer’s solution and then with PBS buffer and then individually transferred into sterile PCR tubes (0.2 mL), each containing 20 μL extraction buffer (17.6 μL nuclease-free dH_2_O, 2 μL 5X PCR buffer, 0.2 μL 1% Tween, and 0.2 μL proteinase K). The buffers with single virgin females were frozen at −20°C for 60 min or overnight and then immediately incubated in a water bath at 65°C for 1.2 h, followed by incubation at 95°C for 10 min. The lysates were cooled on ice and centrifuged at 6500 × g for 2 min. The following primers were used for PCR reactions: the internal transcribed spacer regions (ITS1-5.8S-ITS2) were amplified using primers 18S: (5′-TTGATTACGTCCCTGCCCTTT-3′) (forward), and 28S: (5′-TTTCACTCGCCGTTACTAAGG-3′) (reverse) (Vrain et al., 1992). The D2D3 regions of 28S rRNA were amplified using primers D2F: 5′-CCTTAG TAACGGCGAGTGAAA-3′ (forward) and 536: 5′-CAGC TATCCTGAGGAAAC-3′ (reverse) (Nadler et al., 2006). The 12S mitochondrial gene was amplified using the primers 505F: 5′-GTTCCAGAATAATCGGCTAGAC-3′ (forward) and 506R: 5′-TCTACTTTACTACAACTTACT CCCC-3′ (reverse) (Nadler et al., 2006) and the cytochrome oxidase subunit I (COI) gene was amplified using the universal primers LCO-1490 (5′-GGTCAACAAA TCATAAAGATATTGG-3′) (forward) and HCO-2198 (5′-TAAACTTCAGGGTGACCAAAAAATCA-3′) (reverse) (Folmer et al., 1994). The 25 µL PCR reactions consisted of 12.5 µL of Dream Taq Green PCR Master Mix (Thermo Scientific, USA), 0.5 µL of each forward and reverse primer at 10 µm, 2 µL of DNA extract and 9.5 µL of nuclease-free distilled water. The PCR reaction was performed using a thermocycler with the following settings: for ITS and D2-D3 markers, 1 cycle of 5 min at 94°C followed by 37 cycles of 30 sec at 94°C, 30 sec at 50°C, 1 min 30 s at 72°C, and by a single final elongation step at 72°C for 10 min. For the 12S marker, the PCR protocol included initial denaturation at 94°C for 3 min, followed by 30 cycles of 94°C for 30 s, 50°C for 30 s, and 72°C for 45 s, followed by a final extension at 72°C for 15 min. For the COI marker, the PCR program was as follows: one cycle of 94°C for 2 min followed by 37 cycles of 94°C for 30 s, 51°C for 45 s, 72°C for 2 min, and a final extension at 72°C for 12 min. PCR was followed by electrophoresis (40 min, 130 V) of 10 μL of PCR products in a 1% TBA (tris–boric acid–EDTA) buffered agarose gel stained with SYBR Safe DNA Gel Stain (Invitrogen, Carlsbad, California, USA) (Bhat et al., 2020b). PCR products were purified using QIAquick PCR Purification Kit (Qiagen, Valencia, CA) and sequenced using reverse and forward primers by Sanger sequencing (Bioserve Ltd., Hyderabad, India). Obtained sequences were manually edited and trimmed using BioEdit and deposited in the NCBI under the accession numbers: OQ40749, OQ407497, and OQ407497 for ITS; OQ407498, OQ407499, and OQ407500 for 28S; OQ404917, OQ407535, and OQ407536 for *mtCOI;* and OQ407491, OQ407492, and OQ407493 for *mt12S*.

To obtain genomic sequences of nematodes that belong to all the validly described species closely related to *S. anantnagense* n. sp., we searched the database of the National Center for Biotechnology Information (NCBI) using the Basic Local Alignment Search Tool (BLAST) (Altschul et al., 1990). *Steinernema monticola* (AB698756, GU395647, AY943994, and AY944020) was used as an outgroup in ITS, D2D3, *mt*COI, and *mt*12S *based* phylogenetic trees. The resulting sequences were aligned with MUSCLE (v3.8.31) (Edgar, 2004) and used to reconstruct phylogenetic relationships by the Maximum Likelihood method based on the following nucleotide substitution models: Hasegawa-Kishino-Yano model (HKY+G) (ITS), Tamura–Nei (TN93+G+I) (D2–D3 & COI), and Tamura 3-parameter (T92+G) (12S). To select the best substitution models, best-fit nucleotide substitution model analyses were conducted in MEGA 11 (Nei & Kumar, 2000; Tamura et al., 2021). The trees with the highest log likelihood are shown. The percentages of trees where the associated taxa clustered together are displayed next to the branches. Initial tree(s) for the heuristic search were obtained automatically by applying Neighbor–Join and BioNJ algorithms to a matrix of pairwise distances estimated using the Maximum Composite Likelihood (MCL) approach, and then selecting the topology with superior log likelihood value. In some cases, a discrete Gamma distribution (+G) was used to model evolutionary rate differences among sites and the rate variation model allowed for some sites to be evolutionarily invariable (+I). The trees are drawn to scale, with branch lengths measured in the number of substitutions per site. Graphical representation and edition of the phylogenetic trees were performed with Interactive Tree of Life (v3.5.1) (Chevenet et al., 2006; Letunic and Bork, 2016).

### Symbiotic relationships

The *Xenorhabdus* entomopathogenic bacteria associated with *S. anantnagense* n. sp. Steiner_6, Steiner_7, and Steiner_8 nematodes were isolated as described previously (Machado et al., 2018; Machado et al., 2019). Briefly, *G. mellonella* (n = 10) larvae were exposed to 100 nematode infective juveniles. Two to three days later, insect cadavers were surface–sterilized with 0.1% sodium hypochlorite solution and cut open with a sharp blade. Sterile polypropylene inoculation loops were inserted into the cadaver, and the loops were then streaked on LB agar plates and incubated at 28°C for 24–48 h. *Xenorhabdus*–like colonies were sub-cultured until monocultures were obtained. The strains were further sub-cultured and maintained on LB agar plates at 28°C. To establish their taxonomic identities, we reconstructed phylogenetic relationships based on whole genome sequences of the isolated bacteria and all the different species of the genus *Xenorhabdus* (Machado et al., 2023) and genomic sequences were obtained as described by Machado et al. (2021). Genome sequences were deposited in the National Centre for Biotechnology Information, and accession numbers are listed in Table S3. Phylogenetic relationships were reconstructed based on the assembled genomes and the genome sequences of all validly published species of the genus with publicly available genome sequences as described by Machado et al. (2023). Whole genome sequence similarities were calculated by the digital DNA-DNA hybridization (dDDH) method using the recommended formula 2 of the genome-to-genome distance calculator (GGDC) web service of the Deutsche Sammlung von Mikroorganismen und Zellkulturen GmbH (DSMZ) (Auch et al., 2010, 2020; Meier-Kolthoff et al., 2013, 2014).

## Results and Discussion

Three populations of *Steinernema* nematodes, Steiner_6, Steiner_7 and, Steiner_8, were isolated from agricultural soils in the Bijbehara area of district Anantnag, India. Analysis of several taxonomically relevant markers show that Steiner_6, Steiner_7, and Steiner_8 are conspecific, belong to the “*Feltiae–Kushidai–Monticola*” superclade or “*Kushidai*” clade, are closely related to *S. akhursti*, *S. populi*, *S. sangi,* and *S. kushidai*, and represent a new species, for which we propose the name *Steinernema anantnagense* n. sp. To describe this new species, we compared this species with other closely related species at the molecular and morphological levels and conducted cross-hybridization and self-crossing experiments. As all three populations are identical at the molecular level, we selected Steiner_7 for in-depth morphological and morphometrical identification.

### Systematics

#### *Steinernema anantnagense* n. sp.

**Figure 1:**
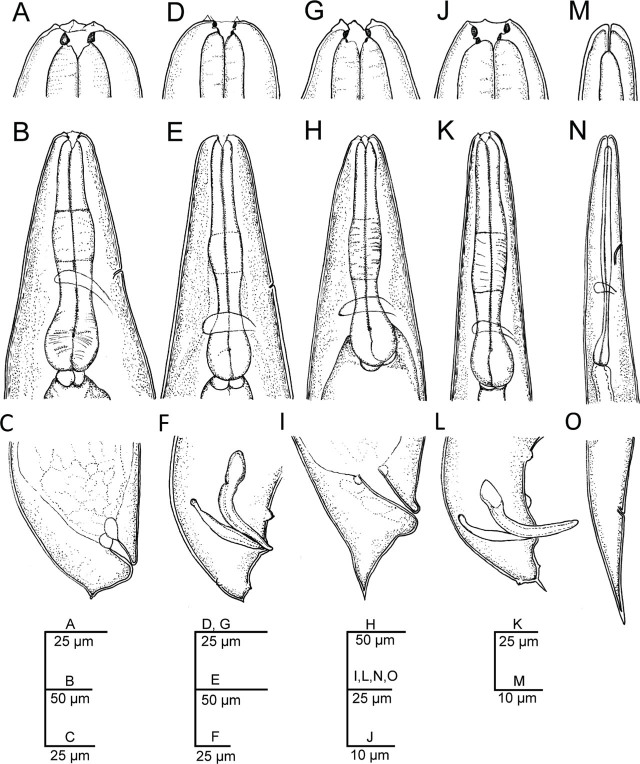
Line drawings of first- and second-generation adults and infective juveniles of *Steinernema anantnagense* n. sp. (A–C) First-generation female: (A) Anterior end; (B) Neck region; (C) Posterior end. (D–F) First-generation male: D) Anterior end; (E) Neck region; (F) Posterior end. (G–I) Second-generation female: (G) Anterior end; (H) Neck region; (I) Posterior end. (J–L) Second-generation male: (J) Anterior end; (K) Neck region; (L) Posterior end. (M–O) Infective juvenile: (M) Anterior end; (N) Neck; (O) Posterior end.

**Figure 2:**
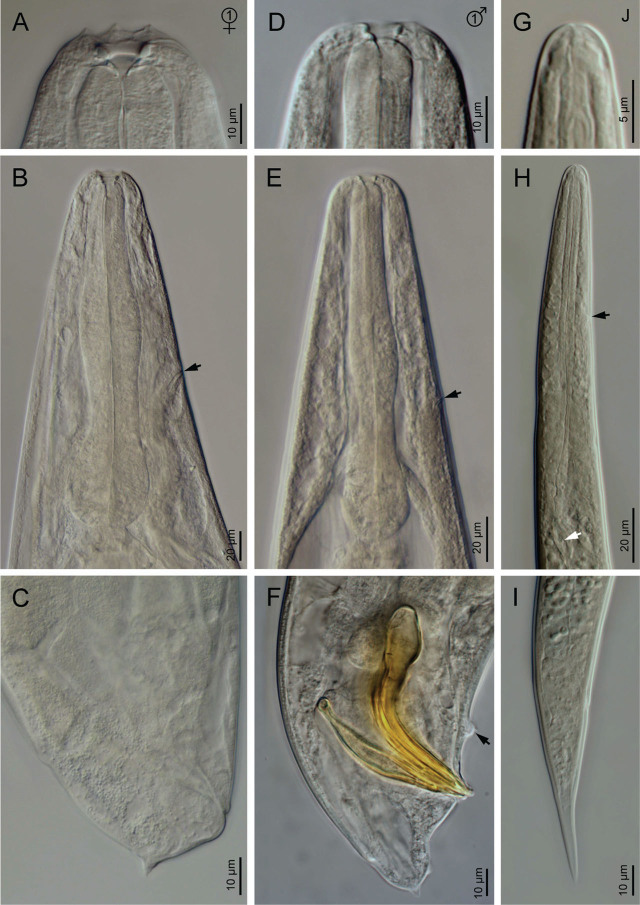
Light microscope micrographs of first-generation adults and IJ of *Steinernema anantnagense* n. sp. (A–C) Female: (A) Anterior end; (B) Neck region (arrow pointing to the excretory pore); (C) Posterior end. (D–F) Male: (D) Anterior end; (E) Neck region (arrow pointing to the excretory pore); (F) Posterior end (arrow pointing to the mid-ventral genital papillae). (G–I) Infective juvenile: (G) Anterior end; (H) Neck region (black arrow pointing to the excretory pore, white arrow pointing to the bacteria sac); (I) Posterior end.

**Figure 3:**
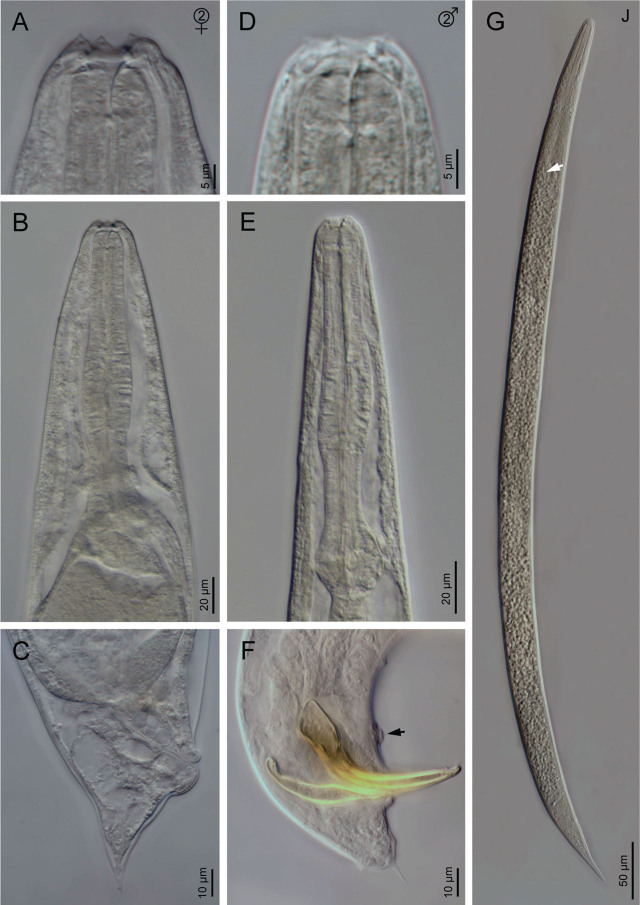
Light microscope micrographs of second-generation adults and IJ of *Steinernema anantnagense* n. sp. (A–C) Second-generation female: (A) Anterior end; (B) Neck region; (C) Posterior end. (D–F) Second-generation male: D) Anterior end; (E) Neck region; (F) Posterior end (arrow pointing to the mid-ventral genital papillae). (G) Entire infective juvenile (arrow pointing to the bacteria sac).

**Figure 4:**
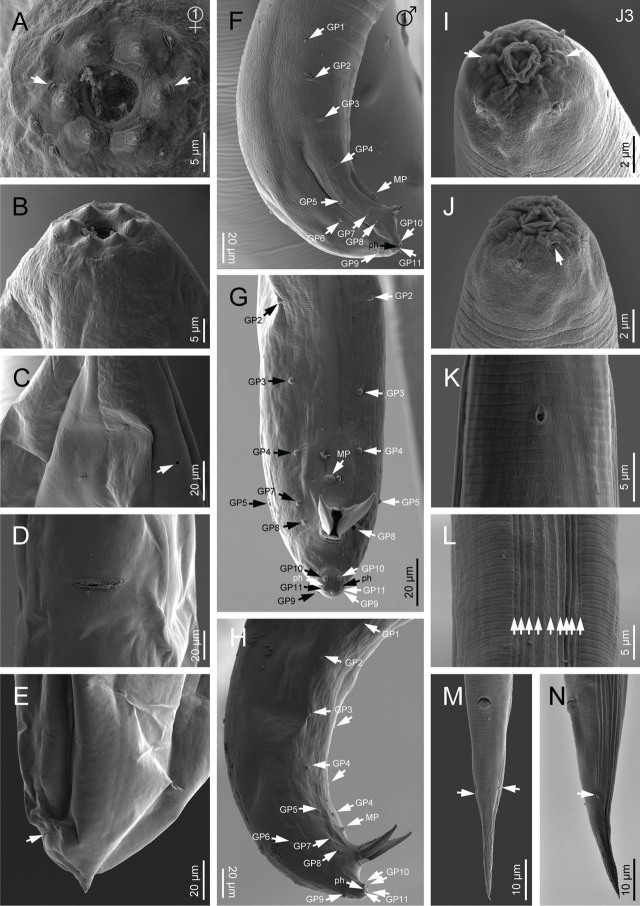
Scanning electron microscope micrographs of first-generation adults of *Steinernema anantnagense* n. sp. (A, B) Lip region of female in frontal and lateral view, respectively (arrows pointing to the amphids); (C) Excretory pore of female; (D) Vulva in ventral view; (E) Female posterior end (arrow pointing to the anus). (F–H) Male posterior end in lateral, ventral and subventral view, respectively (GP: genital papilla, MP: mid-ventral papilla, ph: phasmid). (I, J) Lip region of IJ in frontal and lateral view, respectively (arrows pointing to the amphids); (K) Excretory pore of IJ; (L) Lateral field of IJ; (M, N) Posterior end of IJ in ventral and lateral view, respectively (arrows pointing to the phasmids).

**Figure 5:**
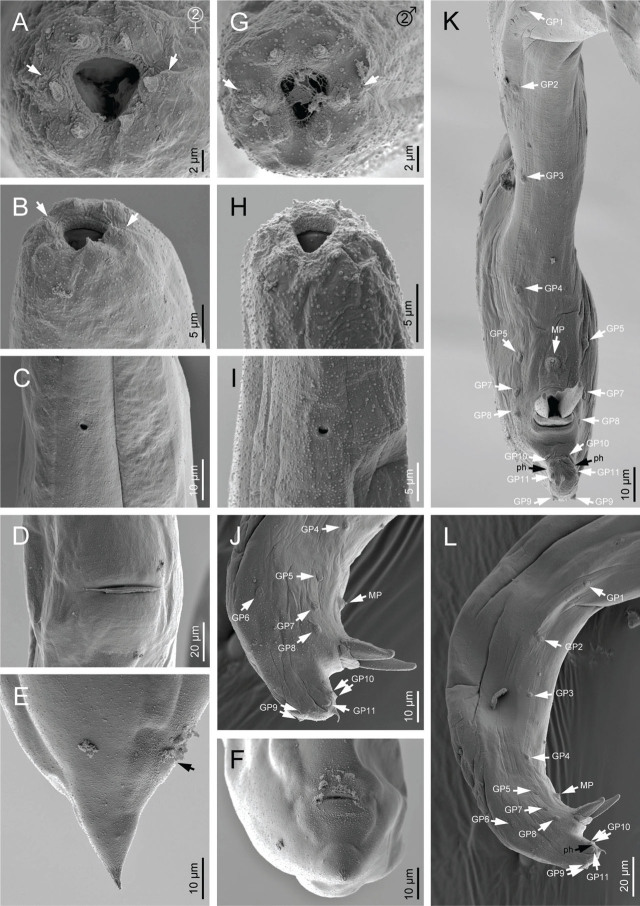
Scanning electron microscope micrographs of second-generation adults and IJ of *Steinernema anantnagense* n. sp. (A, B) Lip region of female in frontal and lateral view, respectively (arrows pointing to the amphids); (C) Excretory pore of female; (D) Vulva in ventral view; (E, F) Female posterior end in lateral and ventral view, respectively (arrow pointing to the anus). (G, H) Lip region of male in frontal and lateral view, respectively (arrows pointing to the amphids); (I) Male excretory pore; (J–L) Male posterior end in lateral, ventral and subventral view, respectively (GP: genital papilla, MP: mid-ventral papilla, ph: phasmid).

(Figs. 1–5, Table 1)

**Table 1.** Morphometrics of the IJs and adult generations of *Steinernema anantnagense* n. sp. (Steiner_7). All characters are in µm (except n, ratios and percentages) and given as mean ± s.d. (range).

**Characters**		**First Generation**		**Second Generation**		**Infective Juveniles (paratypes)**
	
	**Male (holotype)**	**Male (paratypes)**	**Female (paratypes)**	**Male (paratypes)**	**Female (paratypes)**	
n	1	20	20	20	20	20
Body length (L)	1279	1618 ± 246 (1223–1899)	3765 ± 441 (2327–4872)	1068 ± 76 (899–1168)	2081 ± 200 (1761–2437)	789 ± 35 (749–834)
a (L/BD)	6.6	8.3 ± 1.1 (6.4–9.8)	23 ± 1.2 (17–29)	19 ± 2.6 (15.1–25.3)	14.7 ± 1.5 (12.1–17.4)	22 ± 1.9 (19–24)
b (L/NL)	7.0	9.2 ± 1.4 (7.0–11.3)	17.0 ± 2.4 (13–21)	7.1 ± 0.6 (5.9–8.2)	11.0 ± 1.0 (9.6–13.5)	6.0 ± 0.4 (5.5–6.7)
c (L/T)	35	49 ± 9.0 (34–64)	100 ± 18 (61–122)	43 ± 5.8 (31–52)	54 ± 6.6 (43–67)	13.8 ± 1.8 (12.2–16.4)
c’ (T/ABW)	1.2	1.1 ± 0.2 (0.8–1.6)	0.7 ± 0.1 (0.5–0.8)	0.9 ± 0.2 (0.6–1.4)	1.0 ± 0.1 (0.8–1.3)	1.8 ± 0.2 (1.6–2.1)
V (VA/L×100)		–	53 ± 1.7 (50–58)	–	53 ± 1.7 (50–57)	–
Max. body diam. (MBD)	193	194 ±11.3 (167–211)	434 ± 30 (314–409)	57 ± 7.3 (42–63)	143 ± 16 (123–173)	37 ± 3.6 (32–42)
Lip region width	10.2	10.1 ± 1.9 (8.2–12.2)	14.4 ± 1.9 (11.1–18.3)	8.4 ± 1.6 (6.1–12.1)	11.4 ± 1.7 (8.9–14.6)	4.6 ± 2.0 (3.7–5.8)
Stoma length	12.3	13.8 ± 1.6 (10.1–16.9)	16.4 ± 2.4 (12.4–22.1)	11.7 ± 1.1 (10.1–14.1)	14.1 ± 2.3 (11.1–18.1)	10.7 ± 2.0 (8.6–13.8)
Procorpus length	66	57 ± 4.1 (51–66)	65 ± 6.0 (55–76)	47 ± 3.9 (41–53)	54 ± 3.8 (49–65)	43 ± 5.4 (37–52)
Metacorpus length	33	34 ± 1.7 (30–36)	44 ± 4.6 (38–50)	30 ± 2.4 (25–35)	37 ± 2.4 (33–43)	26 ± 2.7 (23–29)
Isthmus length	40	42 ± 4.0 (37–50)	53 ± 6.4 (45–68)	37 ± 1.5 (35–40)	49 ± 3.7 (43–55)	36 ± 2.8 (31–39)
Bulb length (BL)	31	30 ± 2.2 (25–35)	43 ± 4.5 (33–50)	25 ± 2.7 (21–30)	35 ± 2.9 (30–39)	17 ± 1.8 (16–20)
Bulb width (EBW)	26	25 ± 1.6 (21–27)	35 ± 3.5 (27–41)	21 ± 1.9 (18–25)	28 ± 2.7 (25–33)	11 ± 1.7 (9.1–13.9)
Pharynx length (PL)	170	162 ± 5.9 (150–172)	206 ± 14 (182–232)	139 ± 5.0 (129–146)	175 ± 6.4 (164–191)	121 ± 8.0 (109–133)
Nerve ring – ant. end (NR)	109	112 ± 6.8 (103–129)	165 ± 12.1 (143–182)	91 ± 7.0 (75–104)	109 ± 11.9 (88–127)	63 ± 8.4 (54–71)
Excretory pore – ant. end (EP)	114	111 ± 10 (88–124)	112 ± 11.9 (92–140)	80 ± 9.2 (62–98)	92 ± 8.1 (82–112)	55 ± 6.7 (45–62)
Width at excretory pore (WEP)	58	61 ± 3.4 (49–65)	137 ± 11.3 (123–170)	35 ± 3.6 (30–42)	60 ± 6.7 (51–74)	22 ± 2.4 (19–25)
Neck length (stoma+pharynx, NL)	182	176 ± 6.4 (165–185)	223 ± 14 (196–255)	151 ± 5.0 (142–159)	189 ± 6.5 (177–204)	132 ± 8.3 (120–143)
Body width at neck base	79	86 ± 5.8 (76–96)	217 ± 23 (195–264)	41 ± 3.9 (36–50)	95 ± 8.5 (82–107)	29 ± 2.9 (24–32)
Testis reflexion	630	631 ± 37 (576–689)	–	522 ± 32 (481–568)	–	–
Vagina length	–	–	31 ± 3.1 (24–36)	–	24 ± 3.3 (19–30)	–
Body width at vulva	–	–	386 ± 53 (309–498)	–	164 ± 25 (124–193)	–
Vulva – ant. end (VA)	–	–	1989 ± 214 (1247–2252)	–	1105 ± 101 (961–1295)	–
Vulva – post. end (VP)	–	–	1776 ± 242 (1080–2038)	–	976 ± 110 (801–1161)	–
Rectum length	–	–	35 ± 4.0 (29–44)	–	21 ± 2.5 (17–24)	13.8 ± 1.9 (11–16)
Anal body diam. (ABD)	31	32 ± 3.9 (25–36)	86 ± 7.1 (77–110)	29 ± 4.1 (21–35)	41 ± 6.8 (31–53)	20 ± 1.7 (18–22)
Tail length (T)	37	34 ± 2.4 (29–39)	38 ± 4.2 (32–49)	26 ± 3.9 (21–35)	49 ± 3.9 (38–53)	58 ± 6.7 (49–66)
Hyaline part of tail (H)	–	–	–	–	–	16.3 ± 4.1 (11.8–23.7)
Spicule length (SL)	66	64 ± 4.6 (56–70)	–	44 ± 2.6 (40–49)	–	–
Gubernaculum length (GL)	34	36 ± 3.8 (31–43)	–	25 ± 2.3 (21–29)	–	–
Stoma length/lip region width	1.2	1.4 ± 0.2 (0.9–1.8)	1.2 ± 0.2 (0.9–1.8)	1.4 ± 0.3 (0.9–1.9)	1.3 ± 0.3 (0.9–1.8)	2.4 ± 0.5 (1.6–3.0)
Nerve ring % (NR/NL×100)	60	64 ± 5.3 (56–78)	74 ± 7.1 (64–86)	61 ± 4.6 (52–68)	58 ± 6.6 (46–67)	48 ± 7.8 (38–58)
Excretory pore % (EP/NL×100)	62	63 ± 6.1 (50–74)	50 ± 4.6 (42–60)	53 ± 6.6 (41–68)	49 ± 4.1 (43–58)	42 ± 4.4 (35–48)
Rectum% (R/ABD×100)	–	–	0.4 ± 0.1 (0.3–0.6)		0.5 ± 0.1 (0.3–0.8)	0.7 ± 0.1 (0.6–0.9)
D% (EP /NL×100)	62	63 ± 6.1 (49–74)	50 ± 4.9 (43–60)	53 ± 6.6 (41–68)	49 ± 4.1 (43–58)	42 ± 4.4 (35–48)
E% (EP/T×100)	311	333 ± 43 (256–403)	5.7 ± 1.2 (4.5–9.5)	319 ± 59 (219–460)	240 ± 36 (201–362)	96 ± 12.8 (74–113)
SW% (SL/ABD×100)	217	208 ± 39 (154–297)	–	2.3 ± 0.4 (1.8–3.4)		
GS% (GL/SL×100)	51	57 ± 6.7 (46–70)	–	0.6 ± 0.1 (0.4–0.7)		
H% (H/T ×100)	–	–	–	–	–	28 ± 5.8 (20–36)

– = characters absent.

### First generation females (*n* = 20)

Body 2.3–4.9 mm long, and C-shaped after heat relaxation and fixation. Cuticle with poorly visible annuli, with fine transversal incisures. Lateral fields absent. Labial region rounded, and continuous with the adjacent part of the body. Labial plate with six lips that are fused together, each with one labial papilla at the tip and one lower cephalic papilla each except for the lateral lips. Amphid openings present at the lateral lips, close to the labial papilla, with a small transversal slit. Stoma funnel-shaped, shallow, short, and wider at the anterior part. Cheilostom short with rounded and refringent rhabdia; gymnostom scarcely developed with a minute rhabdia; stegostom robust, slightly wider than long, with a funnel-shaped lumen and walls with very minute rhabdia. Pharynx muscular with a subcylindrical procorpus, a somewhat swollen metacorpus, a short and robust isthmus, and a spheroid basal bulb with reduced valves. Nerve ring surrounds the posterior part of the isthmus. Secretory-excretory pore circular, located at the anterior part of the isthmus. Deirids inconspicuous. Cardia short, conoid, and surrounded by intestinal tissue. Intestine tubular without differentiation, with thinner walls at the anterior end. Reproductive system didelphic, amphidelphic, and ovaries are reflexed in dorsal position. Oviducts well developed with glandular spermatheca, and uteri tubular with numerous uterine eggs. Vagina short with muscular walls, vulva protruding in the form of a transverse slit. Rectum 0.3–0.6 times the anal body diameter, with three small rectal glands. Anus well developed. Tail conoid, shorter than body anal diameter, with an acute terminus. Phasmids located at the posterior part of the tail, at 25–30% of the tail length.

### Second generation females (*n* = 20)

Similar to first generation females, but smaller, measuring 1.8–2.4 mm in length. Tail conoid, with an acute terminus, longer than the first generation females.

### First generation males (*n* = 20)

Body 1.2–1.9 mm long, ventrally curved posteriorly, C- or J-shaped when heat killed. General morphology similar to that of females. Reproductive system monorchic, with the testis ventrally reflexed. Spicules paired, symmetrical, ventrally curved with a well-developed manubrium, either rounded or spoon-shaped. Calamus short and narrower, lamina ventrad curved at the anterior part and bears longitudinal ribs, ending in a blunt terminus. Velum indistinct, does not reach the spicule tip, and with no rostrum or retinaculum. Gubernaculum with a rounded manubrium, a fusiform corpus and a narrower and elongated tip, 0.5–0.7 times spicules length. Tail conoid with a rounded terminus bearing a fine mucron. Bursa absent. 11 pairs of genital papillae and a single mid-ventral papilla present, arranged as follows: five pairs (GP1-GP5) subventral precloacal, one pair (GP6) lateral precloacal, one single (MP) midventral precloacal, two pairs (GP7-GP8) sub-ventral ad-cloacal, one pair (GP9) subdorsal postcloacal and two pairs (GP10-GP11) postcloacal at terminus. Phasmids terminal, located laterally between the last pair of genital papillae.

### Second generation males (*n* = 20)

Morphology of second generation males similar to that of the first generation males, but smaller, 0.8–1.2 mm in length. Tail with long, straight and robust mucron. Spicules curved ventrally, with a rhomboid shaped manubrium, slightly broader than the calamus, and a lamina curved ventrally at the anterior part. Ventral velum very reduced, and two longitudinal lateral ribs present. Gubernaculum with a slightly ventrad curved manubrium that is rounded and ventrad bent, slightly fusiform corpus and a narrower and slender terminus. Arrangement of genital papillae and phasmids similar to that of first generation males.

### Infective juvenile (L3 stage) (*n* = 20)

IJ body 0.7–0.8 mm long, almost straight or slightly curved body shape when heat-killed. Body tapers gradually at both extremes, cuticle with transverse incisures, well-developed annuli. Lateral fields begin as a single line close to the anterior end, and increase to eight ridges before gradually reducing to five and then two near the anus and phasmid levels, respectively. Lip region slightly narrower than the adjacent part of the body, six amalgamated lips; with smaller lateral lips, six reduced labial and four prominent cephalic papillae. Amphidial apertures pore-like, oral opening triangular with a noticeable margin. Stoma reduced and tubular with a small lumen, consisting of a short cheilostom and an elongated gymno-stegostom. Pharynx elongated and narrow, with a very long corpus; a slightly narrower isthmus, and a pyriform basal bulb with reduced valves. Nerve ring surrounds the isthmus, excretory pore located at the metacorpus level. Hemizonid present. Deirids inconspicuous. Cardia conoid. Intestine bears a bacterial sac at the anterior part. Rectum long, almost straight, with very short cuticular and elongated cellular parts, anus distinct. Genital primordium located at the equatorial region, tail conoid, tapering gradually to a pointed terminus, with a longer cellular part than the hyaline part and an irregular cellular-hyaline junction. Phasmids located at 37–45% of the tail length.

### Life cycle

*Steinernema anantnagense* n. sp. is a highly pathogenic nematode species that can be easily reared on *G. mellonella* larvae at a temperature ranging from 18–24°C. The life cycle of this new species is similar to the life cycle of other *Steinernema* species. When *G. mellonella* larvae are exposed to 50–100 infective juveniles (IJs), they die within 24–48 h. The first- and second-generation adults of *S. anantnagense* n. sp. can be found in the insect cadavers 3–4 and 5–6 days after infection, respectively. The pre-infective juveniles leave the host body, mature for a few days, and then migrate to the water traps after 10–15 days.

### Type host and locality

The type hosts of *Steinernema anantnagense* n. sp. are unknown as the nematodes of this genus can infect different species of insects, and were obtained from soil samples using the insect baiting technique (Bedding and Akhurst 1975; White 1927). *Steinernema anantnagense* n. sp. Steiner_6, Steiner_7, and Steiner_8 nematodes were isolated, using the *Corcyra cephalonica* baiting method, from soil samples collected around the roots of willow, walnut, and apple trees in the Anantnag district of the Union Territory of Jammu and Kashmir, India (GPS coordinates: Lat. 33.828914°, Long. 75.100091°, 1606 m above sea level).

### Type material

The type material for *Steinernema anantnagense* n. sp. are Steiner_7 nematode populations. For each stage (holotype and paratypes), including first-generation males and females, second-generation males and females, and infective juveniles, six permanent slides were prepared and deposited in the National Nematode Collection of India, located at the Indian Agricultural Research Institute (IARI) in New Delhi, India. Additionally, some permanent slides (paratypes) (n = 15) were deposited at the nematode collection of the Department of Animal Biology, Plant Biology and Ecology at the University of Jaén in Spain (IND001-01 – IND001-15). Live cultures of these nematodes are maintained at the Division of Entomology, Faculty of Agriculture, Sher-e-Kashmir University of Agricultural Science & Technology of Kashmir, India.

### Etymology

The species name is derived from the location Anantnag, a District in the Union Territory of Jammu and Kashmir, India, where the nematode specimens used in this study to describe the new species were obtained.

### Cross-hybridization experiments

Mating experiments were carried out to determine the reproductive isolation of *S. anantnagense* n. sp. by pairing males and females of this species with individuals from other *Steinernema* species, including *S. ichnusae, S. litorale, S. weiseri, S. akhursti, S. citrae, S. cholashanense, S. feltiae, S. silvaticum, S. africanum, and S. xueshanense*. No offspring were produced when *S. anantnagense* n. sp. nematodes were allowed to interact with nematodes of the any of the above mentioned species, indicating that *S. anantnagense* n. sp. is reproductively isolated. Cross tests were also conducted between males and females of Steiner_6, Steiner_7, and Steiner_8 to determine their conspecific status. The results showed that fertile offspring were produced, confirming that they belong to the same species. Controls were also carried out, which included self-crossed species, and offspring were observed in all of them. However, no progeny were observed in the single-female control plates.

### Diagnosis and relationships of *Steinernema anantnagense* n. sp.

*Steinernema anantnagense* n. sp. is characterized by adults with a short stoma, rounded cheilorhabdia, and a robust pharynx with a round basal bulb. Females of the first generation are between 2.3–4.9 mm in length, with didelphic-amphidelphic reproductive system, and possess a shorter conoid tail bearing a short mucron in the first generation (c = 61–122, c′ = 0.5–0.8) and a longer conoid tail with thin mucron in the second generation (c = 43–67, c′ = 0.8–1.3). Males are smaller, between 1.2–1.9 mm in length, with a reproductive system that is monorchid and that has ventrally curved spicules bearing rounded manubrium in the first generation and rhomboid manubrium in the second generation, gubernaculum is fusiform in the first generation and anteriorly ventrad bent in the second generation, tail is conoid and slightly ventrally curved with a minute mucron in the first generation (c = 34–64, c′ = 0.8–1.6) and with a longer and more robust mucron in the second generation (c = 31–52, c′ = 0.6–1.4). The infective juveniles have a nearly straight body (0.7–0.8 mm length), poorly developed stoma and pharynx, lateral fields with eight longitudinal ridges and a conoid-elongate tail (49–66 µm, c = 12–16, c′ = 1.6–2.1) with a hyaline posterior part.

*Steinernema anantnagense* n. sp. belongs to a group of species known as the “*Kushidai-*clade”, and presents several traits common to this group. Several of the morphological and morphometric traits of the IJs and adults overlap with those of other species in the “*Kushidai*-clade”. However, several distinct morphological and morphometrical characteristics can differentiate *S. anantnagense* n. sp. from these closely related species (Tables 2–4).

**Table 2.** Comparison of morphometrics of the third-stage infective juveniles of *Steinernema anantnagense* n. sp. with other members of “*Feltiae-Kushidai*” clade. Measurements are in μm except n, ratio and percentage. Data for new species is in bold.

**Species**	**Reference**	**Country**	**n**	**L**	**BD**	**EP**	**NR**	**NL**	**T**	***a***	***b***	***C***	***c′***	**D%**	**E%**	**H%**
*S. akhursti*	Qiu et al. (2005)	China	20	770–835	33–35	55–60	83–95	115–123	68–75	23–26	6.6–7.2	10–12	3.3–3.7	45–50	73–86	49–56
***S. anantnagense n. sp.***	**Present Study**	**India**	**20**	**749–834**	**32–42**	**45–62**	**54–71**	**120–143**	**49–66**	**19–24**	**5.5–6.7**	**12–16**	**1.6–2.1**	**35–48**	**74–113**	**20–36**
*S. africanum*	Machado et al. (2022)	Rwanda	15	690–802	25–32	54–68	87–132	123–167	52–72	23–30	4.3–6.3	10–15	2.9–4.2	34–46	79–129	28–39
*S. cholashanense*	Nguyen et al. (2008)	China	20	727–909	26–35	59–65	72–97	110–138	60–80	24–34	6.1–7.2	10–14	3.5–5.0	46–53	76–91	33–47
*S. citrae*	Stokwe et al. (2011)	South Africa	20	623–849	23–28	49–64	83–108	118–137	63–81	25–34	5.1–7.1	13–14	13–17	39–58	85–132	37–50
*S. feltiae*	Nguyen et al. (2007)	Russia	25	766–928	22–32	58–67	108–117	130–143	81–89	27–34	5.8–6.8	9.4–11	4.5–5.1	44–50	67–81	37–51
*S. hebeiense*	Chen et al. (2006)	China	20	610–710	23–28	43–51	73–83	100–111	63–71	24–28	5.7–6.7	9.4–11	NA	40–50	65–80	32–50
*S. ichnusae*	Tarasco et al. (2008)	Italy	20	767–969	27–35	59–68	94–108	119–148	76–89	24–32	5.6–6.9	9–12	4.2–5.1	42–49	68–83	44–50
*S. jollieti*	Spiridonov et al. (2004)	USA	12	625–820	20–28	53–65	NA	115–135	60–73	25–34	4.9–6.4	9–12	NA	46–50	NA	46–60
*S. kraussei*	Nguyen et al. (2007)	Germany	25	797–1102	30–36	50–66	99–111	119–145	63–86	NA	NA	NA	NA	NA	NA	35–40
*S. kushidai*	Mamiya (1998)	Japan	20	424–662	22–31	42–50	70–84	106–120	44–59	19–25	4.9–5.9	10–13	NA	38–44	NA	NA
*S. litorale*	Yoshida (2004)	Japan	25	834–988	28–33	54–69	89–104	114–133	72–91	27–31	6.7–7.9	10–11.9	3.8–5.4	44–56	68–84	NA
*S. nguyeni*	Malan et al. (2016)	South Africa	20	673–796	22–28	47–58	74–86	101–121	61–73	27–33	6.2–7.4	10–12	2.8–4.8	43–57	70–86	20–31
*S. oregonese*	Liu and Berry (1996)	USA	20	820–1110	28–38	60–72	NA	116–148	64–78	24–37	6–8	12–16	NA	40–60	90–110	30–33
*S. populi*	Tian et al. (2022)	China	25	973–1172	33–41	70–86	98–113	134–159	55–72	28–33	6.8–7.5	15–20	2.4–3.3	47–61	105–140	26–44
*S. puntauvense*	Uribe-Lorío et al. (2007)	Costa Rica	19	631–728	31–38	20–30	46–69	81–103	51–59	17–23	7.1–7.9	11–13	NA	25–50	35–56	52–55
*S. sandneri*	Lis et al. (2021)	Poland	25	708–965	23–32	44–64	83–118	123–151	64–86	27–34	5.5–6.9	11–13	NA	36–45	63–86	23–40
*S. sangi*	Phan et al. (2001)	Vietnam	20	704–784	30–40	46–54	78–97	120–138	76–89	19–25	5.6–6.3	9–10	NA	36–44	56–70	44–52
*S. silvaticum*	Sturhan et al. (2005)	Germany	26	670–975	26–35	51–73	75–109	100–141	63–86	23–33	6.3–7.7	10–13	3.1–4.9	46–56	–	37–53
*S. tielingense*	Ma et al. (2012)	China	20	824–979	32–38	64–73	90–105	120–135	74–85	27–31	6.7–7.9	10–12	3.5–4.6	44–56	68–84	53–64
*S. texanum*	Nguyen et al. (2007)	USA	20	732–796	29–34	52–62	84–102	111–120	60–79	22–27	6.2–7.0	10–13	3.3–4.6	46–53	76–88	53–69
*S. xinbinense*	Ma et al. (2012)	China	20	635–744	28–31	46–53	75–90	109–125	65–78	21–25	5–7	8–11	3–5	40–47	65–78	30–42
*S. xueshanense*	Mrácek et al. (2009)	China	20	768–929	29–33	60–72	81–96	130–143	80–92	26–32	5.8–7.0	9–11	3.8–5.1	46–52	70–90	46–55
*S. weiseri*	Mrácek et al. (2003)	Czech Republic	20	586–828	24–29	43–65	72–92	95–119	49–68	25–33	5.7–7.2	10–14	3.2–4.1	44–55	NA	18–24

NA = Not available; P = Present; A = Absent.

**Table 3.** Comparison of morphometrics of the first-generation males of *Steinernema anantnagense* n. sp. with other members of “*Feltiae-Kushidai*” clade. Measurements are in μm except n, ratio and percentage. Data for new species is in bold.

**Species**	**n**	**L**	**BD**	**EP**	**NR**	**NL**	**T**	**SL**	**GL**	***a***	***B***	***c***	***c′***	**D%**	**SW%**	**GS%**	**Mucron**
*S. akhursti*	20	1350–1925	115–150	93–113	120–163	168–205	30–40	85–100	58–68	NA	NA	NA	NA	52–61	140–200	65–77	P
***S. anantnagense* n. sp.**	**20**	**1223–1899**	**167–211**	**88–124**	**103–129**	**165–185**	**29–39**	**56–70**	**31–43**	**6–10**	**7–11**	**34–64**	**0.8–1.6**	**49–74**	**154–297**	**46–70**	**P**
*S. africanum*	15	977–1400	65–131	69–109	79–104	132–147	34–46	65–76	32–49	9–12	7–12	25–34	0.9–1.1	52–74	144–197	49–68	P
*S. cholashanense*	20	1070–1778	73–204	75–135	91–126	135–173	29–43	60–71	32–45	9–24	8–11	36–51	0.6–0.9	50–85	92–144	61–85	P
*S. citrae*	20	1028–1402	87–113	64–92	92–119	123–155	17–31	57–80	32–59	NA	NA	NA	NA	47–67	156–233	48–89	P
*S. feltiae*	25	1414–1817	60–90	110–126	NA	164–180	37–43	65–77	34–47	NA	NA	NA	NA	51–64	99–130	52–61	P
*S. hebeiense*	20	1036–1450	74–98	58–73	78–93	118–132	24–35	51–63	38–50	12–17	8–11	30–49	0.6–0.9	48–59	120–170	60–90	A
*S. ichnusae*	20	1151–1494	73–204	94–108	NA	135–173	33–48	64–67	43–46	20–29	7–9	29–39	0.8–0.9	59–65	120–162	64–69	A
*S. jollieti*	12	1296–1952	98–135	83–110	NA	110–168	24–38	55–70	45–60	12–19	8–14	53–86	NA	53–83	NA	NA	A
*S. kraussei*	20	1200–1600	110–144	73–99	95–122	137–178	36–44	42–53	29–37	11	9 NA	NA	NA	NA	NA	NA	P
*S. kushidai*	20	1200–1900	75–156	71–105	120–137	156–189	30–40	48–72	39–60	NA	NA	NA	NA	42–59	NA	NA	A
*S. litorale*	25	1230–1514	82–111	77–107	94–128	133–163	26–41	67–89	44–64	12–16	8–10	33–56	0.6–0.9	34–56	154–200	62–81	P
*S. nguyeni*	20	818–1171	58–106	47–71	70–103	112–144	18–25	58–75	30–55	11–15	7–10	38–53	0.6–0.8	38–57	185–279	46–81	P
*S. oregonense*	20	1560–1820	105–161	95–139	101–133	139–182	24–32	65–73	52–59	NA	NA	NA	0.6 NA	64–75	NA	NA	A
*S. populi*	25	1258–1514	66–95	95–121	107–143	131–177	39–68	57–77	38–60	15–20	8–10	20–33	0.8–1.5	59–78	107–160	58–82	P/A
*S. puntauvense*	19	1010–1931	101–139	68–114	104–128	130–159	28–40	71–81	30–40	NA	NA	NA	NA	45–85	140–200	55–75	P
*S. sandneri*	25	1206–1635	124–178	64–92	112–138	148–170	35–46	53–65	39–50	9–11	8–10	31–42	NA	42–59	97–127	61–83	P
*S. sangi*	20	1440–2325	120–225	67–99	109–166	150–221	27–42	58–80	34–46	NA	NA	NA	NA	42–63	120–160	50–70	P
*S. silvaticum*	26	975–1270	52–78	71–92	90–126	116–168	20–47	42–64	30–43	14–20	8–9	24–55	0.8–1.4	45–63	NA	NA	P
*S. tielingense*	20	1430–2064	111–159	94–133	96–132	145–173	22–33	79–98	49–70	11–18	9–13	57–85	0.3–0.6	64–78	176–212	59–82	A
*S. texanum*	20	1197–1406	81–116	79–100	94–114	123–147	19–30	55–66	39–53	NA	NA	NA	NA	58–73	127–203	62–84	A
*S. xinbinense*	20	1133–1440	90–126	57–75	91–120	138–159	30–41	49–62	30–41	11–13	7–9	31–39	0.7–1.0	41–50	114–156	54–72	P
*S. xueshanense*	20	1313–2040	97–159	113–137	NA	151–175	29–48	66–91	41–60	NA	NA	NA	NA	73–87	93–172	58–95	A
*S. weiseri*	20	990–1395	84–138	57–84	94–115	134–154	19–32	62–72	46–57	9–12	7–10	36–64	0.6–0.9	39–60	150–240	70–85	A

NA = Not available; P = Present; A = Absent.

**Table 4.** Comparison of morphometrics of the first-generation females of *Steinernema anantnagense* n. sp. with other members of “*Feltiae-Kushidai*” clade. Measurements are in μm except n, ratio and percentage. Data for new species is in bold.

**Species**	**L**	**BD**	**EP**	**NR**	**NL**	**T**	***A***	***B***	***c***	***c′***	**V**	**ABD**	**D%**	**Mucron**
*S. akhursti*	5625–9000	200–270	113–138	150–175	213–258	38–63	30*	32*	149*	0.6*	48–53	68–100	NA	P
***S. anantnagense* n. sp.**	**2327–4872**	**314–409**	**92–140**	**143–182**	**196–255**	**32–49**	**17–29**	**13–21**	**61–122**	**0.5–0.8**	**50–58**	**77–110**	**43–60**	**P**
*S. africanum*	2469–4215	154–194	67–111	79–130	170–201	35–55	13–27	13–24	51–104	0.7–1.0	50–57	37–70	32–62	P
*S. cholashanense*	3232–6363	156–332	111–148	176–223	181–231	46–70	13–23	18–32	62–119	0.6–1.0	50–57	54–105	29–65	P
*S. citrae*	2038–4019	137–212	54–90	130–179	189–220	33–60	NA	NA	NA	NA	50–59	43–79	27–46	P
*S. feltiae*	3095–3774	170–254	68–97 **	70–97 **	197–304	39–70	14–20	12–17	49–88	0.7–1.2 *	44–57	47–62	40–54 *	P
*S. hebeiense*	3972–4254	142–245	48–95	88–123	133–158	133–158	17–25	21–29	67–129	0.5–0.9	50–57	45–65	36–66	A
*S. ichnusae*	4547–6186	242–323	106–156	NA	215–262	51–79	17–24	21–26	68–113	0.6–1.0	51–57	70–94	47–63	P
*S. jollieti*	3746–6030	219–298	96–136	NA	184–310	31–55	15–24	19–31	72–185	NA	44–56	NA	52	P
*S. kraussei*	2500–5400	153–288	66–99	127–146	178–205	33–59	17	22	88	NA	54	39–50	45	P
*S. kushidai*	2100–4700	54–59	78–105	111–144	204–255	30–45	NA	NA	NA	NA	54–59	54–84	37–46	A
*S. litorale*	3930–5048	175–215	65–105	130–165	185–213	25–60	21–26	20–26	78–157	0.5–0.9	0.5–0.9	55–75	33–57	P
*S. nguyeni*	2290–5361	130–216	49–98	84–139	137–194	20–67	15–30	15–30	53–165	0.6–1.1	52–63	130–216	30–56	A
*S. oregonense*	4400–6200	217–268	217–268	129–162	186–220	28–46	NA	NA	NA	NA	46–56	42–79	43–57	A
*S. populi*	4038–13762	217–531	90–178	150–213	213–278	41–88	18–36	19–50	75–182	0.5–0.9	45–60	60–157	36–65	A
*S. puntauvense*	3687–8335	181–221	51–85	123–146	141–206	41–66	NA	NA	NA	NA	51–55	57–102	25–45	P
*S. sandneri*	4244–5014	181–261	61–102	132–158	173–194	32–61	17–25	24–27	75–140	NA	49–57	62–122	36–54	P
*S. sangi*	4830–7200	270–360	80–121	140–170	216–240	36–62	NA	NA	NA	NA	43–530	84–140	35–51	P
*S. silvaticum*	1520–3290	50–175	50–175	50–175	121–188	33–79	15–41	10–18	34–80	1.0–1.8	44–57	26–53	33–79	A
*S. texanum*	2720–3623	130–202	78–107	111–135	160–189	30–52	NA	NA	NA	NA	50–55	50–71	NA	A
*S. tielingense*	4028–8538	200–307	82–103	111–144	186–263	40–69	17–32	21–45	72–158	0.5–0.9	49–54	56–92	32–49	A
*S. xinbinense*	3025–5121	159–200	70–87	106–141	167–192	30–53	19–25	17–26	79–123	0.5–0.8	46–57	50–67	38–45	P
*S. xueshanense*	4181–8181	182–343	117–148	NA	196–274	43–66	NA	NA	NA	NA	52–62	38–72	NA	P
*S. weiseri*	3780–5940	202–263	75–86	108–154	162–226	38–59	17–29	22–31	87–156	0.5–0.8	50–58	51–80	NA	P

NA = Not available; P = Present; A = Absent.

*Steinernema anantnagense* n. sp. and *S. akhursti* (Qiu et al., 2005) morphologically differ in several traits. In the case of IJs, the distance from the head to the nerve ring is shorter in *S. anantnagense* n. sp. (54–71) compared to *S. akhursti* (83–95 μm), and the tail is shorter (49–66 μm) in *S. anantnagense* n. sp. than in *S. akhursti* (68–75 μm). The ratio of body length to tail (c) is greater in *S. anantnagense* n. sp. (12–16) than in *S. akhursti* (10–12), while the ratio of tail to body length (c′) is lower in *S. anantnagense* n. sp. (1.6–2.1) than in *S. akhursti* (3.3–3.7). Additionally, *S. anantnagense* n. sp. has a smaller H% value (20–36) compared to *S. akhursti* (49–56) (Table 2). The first-generation males of *S. anantnagense* n. sp. have a larger body diameter (167–211 μm) and much shorter spicule and gubernaculum (56–70 μm and 31–43 μm, respectively) compared to *S. akhursti* (body diameter: 115–150 μm, spicule: 85–100 μm, gubernaculum: 58–68 μm) (Table 3). The first-generation females of the two species also differ in some morphometric measurements (Table 4).

*Steinernema anantnagense* n. sp. differs from *S. populi* (Tain et al., 2022) in IJ body length (0.75–0.83 vs. 0.97–1.17 mm), the distance from anterior end to excretory pore (45–62 vs. 70–86 μm) and to nerve ring (54–71 vs. 98–113) μm), tail length (49–66 vs. 55–72 μm) and lower a, b, c and c′ ratios and lower D% (Table 2). The first-generation males of the new species differ from those of *S. populi* in body diameter (167–211 vs. 66–95 μm), tail length (29–39 vs. 39–68 μm), lower a and c ratios, and mucron (always present vs. present or absent) (Table 3). The first-generation females of the new species differ from those of *S. populi* in mucron (present vs. absent) and other characters (Table 4).

*Steinernema anantnagense* n. sp. can be distinguished from *S. kushidai* (Mamiya, 1988) by several morphological features. The body length of IJs in *S. anantnagense* n. sp. is longer (0.75–0.83 mm) than in *S. kushidai* (0.42–0.66 mm), and the distance from the anterior end to the nerve ring is shorter (54–71 μm) in *S. anantnagense* n. sp. compared to *S. kushidai* (70–84 μm). Additionally, the neck length is longer (120–143 μm), and the ratio c is higher (12–16) in *S. anantnagense* n. sp. than in *S. kushidai* (neck length: 106–120 μm, c: 10–13) (Table 2). The first-generation males of *S. anantnagense* n. sp. can be distinguished from *S. kushidai* by having a larger body diameter (167–211 vs. 75–156 μm), a shorter tail (29–39 vs. 40 μm), and the presence of a mucron, while *S. kushidai* does not have a mucron (Table 3). The first-generation females of *S. anantnagense* n. sp. also have a larger body diameter (314–409 μm), a longer distance from the anterior end to the nerve ring (143–182 μm), and the presence of a mucron, which are all different from *S. kushidai* (54–59 μm, 111–144 μm, and absent mucron, respectively) (Table 4).

In comparison to *S. sangi* (Phan et al., 2001), *S. anantnagense* n. sp. has a longer body length of IJs (0.75–0.83 vs. 0.70–0.78 mm), a shorter distance from the anterior end to the nerve ring (54–71 vs. 78–97 μm), a shorter tail length (49–66 vs. 76–89 μm), and greater c ratio, greater E%, and lower H% (12–16 vs. 9–10, 74–113 vs. 56–70, and 20–36 vs. 44–52, respectively) (Table 2). There are also minor differences in some characters between the first-generation males and females of the new species and those of *S. sangi*, which are presented in Tables 3 and 4, respectively.

The IJs of *S. anantnagense* n. sp. displays several distinguishing features from other related species. In comparison to *S. cholashanense* (Nguyen et al., 2008), the position of the nerve ring is more anterior (54–71 μm vs. 72–97 μm)), and the c′ ratio is lower (1.6–2.1 vs. 3.5–5.0). When compared to *S. hebeiense* (Chen et al., 2006), *S. anantnagense* n. sp. has a greater body length (0.75–0.83 vs. 0.61–0.71 mm), larger body diameter (32–42 vs. 23–48 μm), a more anterior position of the nerve ring (54–71 μm vs. 73–83 μm), a longer neck length (120–143 vs. 100–111 μm), a lower a ratio (19–24 vs. 24–28), and a higher c ratio (12–16 vs. 9.4–11). When compared to *S. tielingense* (Ma et al., 2012), *S. anantnagense* n. sp. has a shorter body length (0.75–0.83 vs. 0.82–0.98 mm), a more anterior position of the excretory pore and nerve ring (45–62 μm and 54–1 μm, respectively, as opposed to 64–73 μm and 90–105 μm, respectively), a shorter tail (49–66 vs. 74–85 μm), smaller ratios of a, b, and c′ and H% (19–24, 5.5–6.7, 1.6–2.1, and 20–36, respectively, as opposed to 27–31, 6.7–7.9, 3.5–4.6, and 53–64, respectively), but a longer c ratio (12–16 vs. 10–12). Compared to *S. xinbinense* (Ma et al., 2012), *S. anantnagense* n. sp. has a greater body length (0.75–0.84 vs. 0.64–0.74 mm), larger body diameter (32–42 μm vs. 28–31 μm), a smaller distance from the anterior end to the nerve ring (54–71 μm vs. 75–90 μm), a shorter tail (49–66 vs. 65–78 μm), a longer c ratio (12–16 vs. 8–11), a smaller c′ ratio (1.6–2.1 vs. 3–5), and a longer E% (74–113 vs. 65–78). *Steinernema anantnagense* n. sp. can be differentiated from *S. xueshanense* (Mrácek et al., 2009) by a smaller distance from the anterior end to the excretory pore and nerve ring (45–62 vs. 60–72 μm and 54–71 vs. 81–96 μm, respectively), a shorter tail length (49–66 vs. 80–92 μm), lower ratios of a and c′ (19–24 vs. 26–32 and 1.6–2.1 vs. 3.8–5.1, respectively), and lower H% (20–36 vs. 46–55). In addition, the position of the IJs nerve ring in the new species is more anterior (54–71 μm) compared to *S. feltiae* (Nguyen, 2007) (108–117 μm), and it also has a shorter tail length (49–66 vs. 81–89 μm) (Table 3).

### Nematode molecular characterization

The ITS regions of *S. anantnagense* n. sp. (Steiner_6, Steiner_7, and Steiner_8) are each 730 bp in length, consisting of ITS1 (278 bp), 5.8S (157 bp), and ITS2 (295 bp). Compared to other related species, the ITS region of *S. anantnagense* n. sp. shows differences of 19–117 bp, resulting in sequence similarity values of 78–97% (Table 5). Similarly, the D2-D3 expansion segments of the 28S rRNA gene of *S. anantnagense* n. sp. differ from those of other species by 5–35 bp, resulting in sequence similarity values of 95–99% (Table 6). In addition, the mitochondrial COI exhibit differences of 65–90 bp, resulting in 82–87% sequence similarity values, respectively (Table S1). Further, the mitochondrial 12S genes also exhibit 33–82 bp differences, resulting in sequence similarity values of 79–92%, respectively (Table S2). When these sequences were compared with sequences in the NCBI database using BLAST search, we observed that the top hit record for the ITS was 97.24% with *S. akhursti* (DQ375757) from China, for the D2D3 was 99.42% with *S. akhursti* (KF289902) from China, for *mt*COI was 88.23% with *S. sangi* (MF621239) from India, and for *mt*12S rRNA was 92.34% with *S. kushidai* (AP017467) from Japan. Taken together, these observations suggest that *S. anantnagense* n. sp. represents a new taxonomic entity within the “*Kushidai*” clade, as evidenced by the lower sequence similarity scores between this species and all other known species, thus supporting its novel taxonomic status.

**Table 5.** Pairwise distances in base pairs of the ITS rRNA regions among closely related *Steinernema* species and *Steinernema anantnagense* n. sp. Data for new species is in bold.

**Species (ITS rRNA)**	***S. anantnagense* n. sp. OQ407490**	***S. akhursti* DQ375757**	***S. kushidai*AB243440**	***S. cholashanense* EF431959**	***S. oregonense* AY230180**	***S. sangi* AY355441**	***S. texanum* EF152568**	***S. xueshanense* FJ666052**	***S. populi* MZ367621**	***S. jollieti* AY171265**	***S. xinbinense* JN171593**	***S. weiseri* KJ696685**	***S. tielingense* GU994201**	***S. africanum* ON041031**	***S. kraussei* AY230175**	***S. citrae* EU754718**	***S. silvaticum* AY230162**	***S. litorale* AB243441**	***S. ichnusae* EU421129**	***S. nguyeni* KP325084**	***S. feltiae* AY230169**	***S. hebeiense* DQ105794**	***S. monticola* AB698756**
***S. anantnagense* n. sp. OQ407490**		**19**	**53**	**72**	**72**	**72**	**72**	**73**	**74**	**74**	**80**	**83**	**85**	**86**	**86**	**87**	**87**	**90**	**91**	**92**	**101**	**116**	**117**
*S. akhursti* DQ375757	97		51	75	74	73	77	76	65	75	82	86	89	89	90	87	91	93	95	94	102	119	115
*S. kushidai* AB243440	92	93		95	89	96	103	90	94	89	97	104	101	103	108	110	104	107	110	110	122	128	130
*S. cholashanense* EF431959	89	88	85		61	74	58	58	106	69	68	72	71	72	83	70	82	73	74	84	75	112	116
*S. oregonense* AY230180	89	88	86	91		47	51	33	99	19	31	43	46	39	52	42	45	56	49	52	51	94	115
*S. sangi* AY355441	89	88	85	88	93		57	58	98	57	54	43	45	60	53	60	61	48	48	54	47	88	103
*S. texanum* EF152568	89	88	83	91	92	91		53	95	61	60	66	67	59	75	55	69	74	69	77	68	110	118
*S. xueshanense* FJ666052	89	88	86	91	95	91	92		99	41	43	46	54	47	60	44	54	57	53	60	54	86	114
*S. populi* MZ367621	89	90	86	83	84	84	85	84		105	103	110	111	112	109	113	115	117	116	112	124	145	123
*S. jollieti* AY171265	88	88	86	89	97	91	91	94	83		48	52	54	55	64	55	58	63	56	62	58	95	115
*S. xinbinense* JN171593	88	87	85	90	96	92	91	94	84	93		45	50	32	58	40	38	58	57	57	50	92	116
*S. weiseri* KJ696685	87	86	83	89	94	94	90	93	82	92	93		17	59	32	60	65	26	27	34	27	80	114
*S. tielingense* GU994201	87	86	84	89	93	93	90	92	82	92	93	98		63	46	65	66	30	28	47	32	88	117
*S. africanum* ON041031	**87**	86	84	89	94	91	91	93	82	92	95	91	91		64	45	35	72	68	64	62	98	124
*S. kraussei* AY230175	**86**	86	82	87	92	92	88	91	82	90	91	95	93	90		65	69	44	46	19	40	89	120
*S. citrae* EU754718	**86**	86	82	89	94	91	92	94	82	92	94	91	90	93	90		55	73	70	64	60	98	118
*S. silvaticum* AY230162	**86**	86	83	87	93	91	89	92	81	91	95	90	90	95	90	92		76	70	68	68	101	126
*S. litorale* AB243441	**86**	85	82	89	92	93	88	91	80	90	91	96	96	89	93	89	88		38	45	34	79	118
*S. ichnusae* EU421129	**86**	85	82	88	93	93	89	92	81	92	92	96	96	90	93	89	89	94		45	25	87	122
*S. nguyeni* KP325084	**85**	85	82	87	92	92	88	91	81	90	91	95	93	90	97	90	90	93	93		41	86	117
*S. feltiae* AY230169	**84**	84	80	88	92	93	89	92	79	91	92	96	95	91	94	91	90	95	96	94		79	119
*S. hebeiense* DQ105794	**81**	80	78	82	85	86	82	86	75	85	86	88	86	84	86	85	84	88	86	87	88		135
*S. monticola* AB698756	**78**	79	76	78	79	81	78	79	77	79	79	79	78	77	78	78	77	78	77	78	78	74	

Below diagonal: percentage similarity; above diagonal: total character difference.

**Table 6.** Pairwise distances in base pairs of the D2D3 fragment of 28S rRNA regions among closely related *Steinernema* species and *Steinernema anantnagense* n. sp. Data for new species is in bold.

**Species (D2D3 rRNA)**	***S. anantnagense* n. sp. OQ407498**	***S. akhursti* KF289902**	***S. weiseri* FJ165549**	***S. oregonense* GU569055**	***S. puntauvense* EF187018**	***S. feltiae* AF331906**	***S. ichnusae* EU421130**	***S. africanum* OM423154**	***S. kushidai* AF331897**	***S. tielingense* GU994202**	***S. populi* MZ367685**	***S. xueshanense* FJ666053**	***S. kraussei* KC631424**	***S. jollieti* GU569051**	***S. cholashanense* EF520284**	***S. texanum* EF152569**	***S. xinbinense* GU994204**	***S. citrae* MF540676**	***S. silvaticum* KC631426**	***S. sangi* MF620997**	***S. nguyeni* KR815816**	***S. monticola* GU395647**
***S. anantnagense* n. sp. OQ407498**		**5**	**12**	**13**	**13**	**13**	**15**	**16**	**17**	**18**	**18**	**18**	**19**	**19**	**23**	**24**	**25**	**25**	**27**	**30**	**31**	**35**
*S. akhursti* KF289902	**99**		16	16	17	17	17	18	18	22	20	22	22	21	26	27	29	27	28	27	33	38
*S. weiseri* FJ165549	**98**	98		12	5	5	7	6	22	13	18	15	16	9	18	21	19	18	24	32	22	37
*S. oregonense* GU569055	**98**	98	98		11	11	13	14	24	10	27	17	8	17	13	22	14	25	19	33	29	38
*S. puntauvense* EF187018	**98**	98	99	99		0	4	5	23	10	23	16	13	10	15	20	16	17	21	30	21	40
*S. feltiae* AF331906	**98**	98	99	99	100		4	5	23	10	23	16	13	10	15	20	16	17	21	30	21	40
*S. ichnusae* EU421130	**98**	98	99	98	99	99		5	25	14	25	17	15	10	19	20	20	17	25	32	21	40
*S. africanum* OM423154	**98**	98	99	98	99	99	99		21	13	22	19	16	8	18	22	19	12	26	30	15	37
*S. kushidai* AF331897	**98**	98	97	97	97	97	97	97		26	30	26	26	29	28	36	29	30	34	32	33	42
*S. tielingense* GU994202	**98**	97	98	99	99	99	98	98	97		27	20	10	18	12	25	12	22	20	36	26	44
*S. populi* MZ367685	**98**	97	98	96	97	97	97	97	96	96		27	30	27	30	29	31	32	38	40	37	44
*S. xueshanense* FJ666053	**98**	97	98	98	98	98	98	98	97	97	96		20	22	20	25	22	30	28	41	33	45
*S. kraussei* KC631424	**98**	97	98	99	98	98	98	98	97	99	96	97		21	13	27	11	27	16	37	31	45
*S. jollieti* GU569051	**98**	97	99	98	99	99	99	99	96	98	96	97	97		23	24	22	20	31	35	24	42
*S. cholashanense* EF520284	**97**	97	98	98	98	98	98	98	96	98	96	97	98	97		27	10	27	19	38	30	45
*S. texanum* EF152569	**97**	96	97	97	97	97	97	97	95	97	96	97	96	97	96		32	32	37	39	35	45
*S. xinbinense* GU994204	**97**	96	98	98	98	98	97	98	96	98	96	97	99	97	99	96		30	17	39	34	47
*S. citrae* MF540676	**97**	96	98	97	98	98	98	98	96	97	96	96	96	97	96	96	96		37	42	11	49
*S. silvaticum* KC631426	**96**	96	97	98	97	97	97	97	95	97	95	96	98	96	98	95	98	95		41	41	50
*S. sangi* MF620997	**96**	96	96	96	96	96	96	96	96	95	95	94	95	95	95	95	95	94	95		46	46
*S. nguyeni* KR815816	**96**	96	97	96	97	97	97	98	96	97	95	96	96	97	96	95	95	99	95	94		51
*S. monticola* GU395647	**95**	95	95	95	95	95	95	95	94	94	94	94	94	94	94	94	94	93	93	94	93	

Below diagonal: percentage similarity; above diagonal: total character difference.

### Nematode phylogenetic relationships

**Figure 6:**
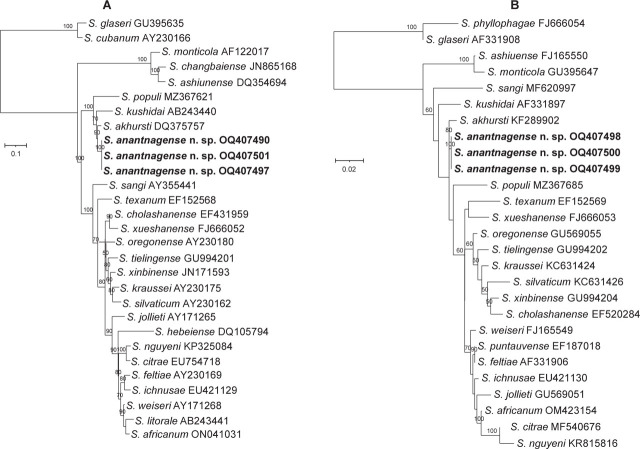
Maximum-likelihood phylogenetic tree between the newly described *Steinernema anantnagense* n. sp. and other closely related species of *Steinernema* species based on nucleotide sequences of: (A) the Internal Transcribed Spacer (ITS1-5.8S-ITS2) rRNA, flanked by primers 18S and 26S, and (B) the D2-D3 expansion segments of the large subunit (28S) of rRNA flanked by primers D2F and 536. Numbers at nodes represent bootstrap values based on 100 replications. Bars represent average nucleotide substitutions per sequence position. NCBI accession numbers of the nucleotide sequences used for the analyses are shown next to the species names. The scale bar shows the number of substitutions per site.

**Figure 7:**
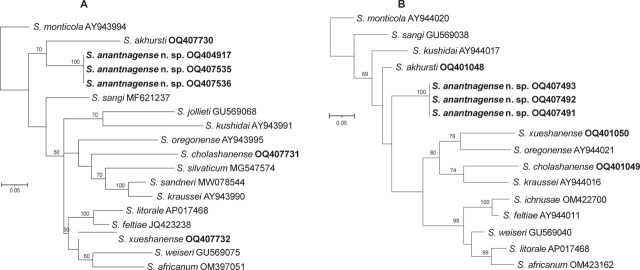
Maximum-likelihood phylogenetic tree between the newly described *Steinernema anantnagense* n. sp. and other closely related species of *Steinernema* species based on the nucleotide sequences of: (A) the COI region of the mitochondrial gene, flanked by primers LCO-1490 and HCO-2198, and (B) the mitochondrial 12S rRNA gene, flanked by primers 505F and 506R. Numbers at nodes represent bootstrap values based on 100 replications. Bars represent average nucleotide substitutions per sequence position. NCBI accession numbers of the nucleotide sequences used for the analyses are shown next to the species names (accession numbers in bold font are the sequences newly generated in this study). The scale bar shows the number of substitutions per site.

Phylogenetic reconstructions based on the nucleotide sequences of the internal transcribed spacer (ITS) marker of the rRNA gene, D2D3 expansion segments of the 28S rRNA gene, the cytochrome oxidase subunit I (COI), and the mitochondrial 12S rRNA gene show that *S. anantnagense* n. sp. Steiner_6, Steiner_7, and Steiner_8 are conspecific and belong to the “*Kushidai*” clade and the “*Feltiae–Kushidai–Monticola*” superclade (Figs. 6 and 7). Phylogenetic analyses of all four abovementioned markers clearly separate *S. anantnagense* n. sp. from all other species. In addition, these phylogenetic reconstructions show that *S. anantnagense* n. sp. is closely related to other Asian species, including *S. akhursti, S. kushidai,* and *S. populi*. No phylogenetic tree was built using the 18S rRNA genetic region because insufficient 18S rRNA gene sequences are publicly available. However, the resulting sequences were deposited in the NCBI databank under the following accession numbers: OQ407498 (Steiner_6), OQ407499 (Steiner_7), and OQ407500 (Steiner_8).

### Symbiotic relationships

**Figure 8:**
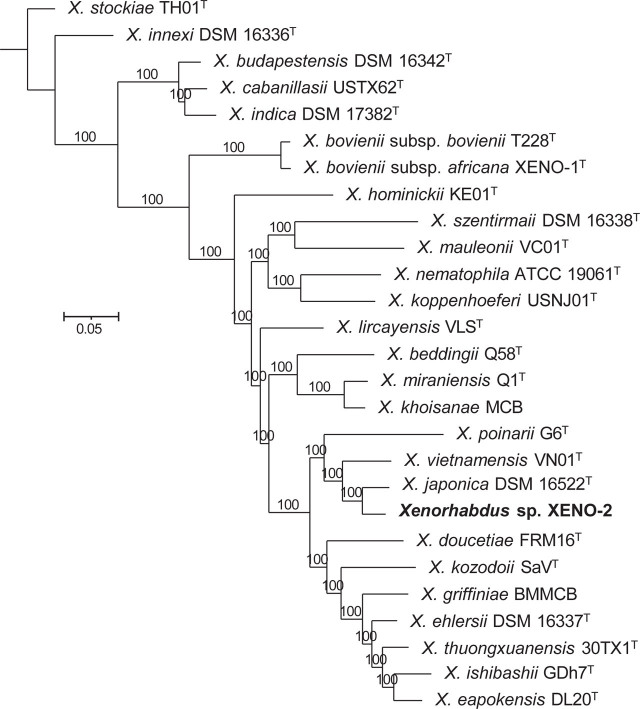
Phylogenetic reconstruction based on core genome sequences of *Xenorhabdus* bacterial strains. 1719910 nucleotide positions (1501 core genes) were used in the analysis. Numbers at the nodes represent SH-like branch supports. Bar represents 0.05 nucleotide substitutions per sequence position. Accession numbers of the genome sequences used for the reconstruction are shown in Table S3.

Phylogenetic reconstructions based on whole genome sequences show that the bacterial symbiont isolated from *S. anantnagense* n. sp. Steiner_7, named here XENO-2, is closely related to *X. japonica* DSM 16522^T^ and *X. vietnamensis* VN01^T^ (Fig. 8). The digital DNA–DNA hybridization (dDDH) values between XENO-2 and *X. japonica* DSM 16522^T^, and between XENO-2 and *X. vietnamensis* VN01^T^ are 51.8% and 40.0%, respectively. These values are below the 70% divergence threshold for prokaryotic species delineation, indicating that XENO-2^T^ represents a novel species within the genus *Xenorhabdus* (Wayne et al., 1987). This species is formally described elsewhere.

### A Side Note on The Nomenclature of *Steinernema Monticolum*

The term “*monticolum*” was introduced by Stock et al. (1997) to refer to the geographic origin of the nematodes studied, which were collected in Mount Jiri (Sancheong, Gyeongnam province, Korea). This term is a combination of “*monti*” referring to “*mountain*” and “*colum*” derived from the Latin suffix “*cola*” meaning “that lives in a place.” However, it should be noted that, as the suffix “*cola*” is a masculine noun in Latin, it does not have gender variations. Therefore, the correct term to use is “*monticola.*” The correct usage of this term has been discussed in detail by Nicolson, 1987. In light of this, we propose to refer to this species as *Steinernema monticola*, as was first used by Choo et al. (1998).

## Conclusions

The differences in morphology, morphometry, molecular characteristics, reproductive isolation, and clear phylogenetic distinction support that Steiner_6, Steiner_7, and Steiner_8 represent a new species of entomopathogenic nematodes. We propose to name this species *Steinernema anantnagense* n. sp. This discovery marks the second new species description in the *Steinernema* genus from the Indian Subcontinent. Our findings provide valuable insights into the biodiversity and distribution of these biological control agents. Furthermore, our results underscore the importance of accurately characterizing newly described *Steinernema* species through the inclusion of all three standard rDNA markers (ITS, SSU, and LSU) in combination with the mitochondrial COI gene, in addition to classical taxonomy. We recommend that all future species descriptions follow this approach.
